# Genetic Polymorphisms of NLRP3 (rs4612666) and CARD8 (rs2043211) in Periodontitis and Cardiovascular Diseases

**DOI:** 10.3390/biology10070592

**Published:** 2021-06-27

**Authors:** Jaideep Mahendra, Abirami Nayaki Rao, Little Mahendra, Hytham N. Fageeh, Hammam Ibrahim Fageeh, Thodur Madapusi Balaji, Saranya Varadarajan, Raghunathan Jagannathan, Deepak Mehta, Venkata V. Suresh, A. Thirumal Raj, Shankargouda Patil

**Affiliations:** 1 Department of Periodontology, Meenakshi Ammal Dental College and Hospital, Chennai 600095, India; abirami.nayaki1791@gmail.com; 2 Maktoum Bin Hamdan Dental University College, Dubai 213620, United Arab Emirates; littlemahendra24@gmail.com; 3 Department of Preventive Dental Science, College of Dentistry, Jazan University, Jazan 45142, Saudi Arabia; hfageeh@jazanu.edu.sa (H.N.F.); hafageeh@jazanu.edu.sa (H.I.F.); 4 Department of Periodontology, Tagore Dental College and Hospital, Chennai 600127, India; tmbala81@gmail.com (T.M.B.); doctorraghunathan@gmail.com (R.J.); 5 Department of Oral Pathology and Microbiology, Sri Venkateswara Dental College and Hospital, Chennai 600130, India; vsaranya87@gmail.com (S.V.); thirumalraj666@gmail.com (A.T.R.); 6 Department of Restorative Dentistry, Tohoku University Graduate School of Dentistry, Miyagi 980-8575, Japan; deepak.mehta.e6@tohoku.ac.jp (D.M.); venkataiah.venkata.suresh.e5@tohoku.ac.jp (V.V.S.); 7 Department of Maxillofacial Surgery and Diagnostic Sciences, Division of Oral Pathology, College of Dentistry, Jazan University, Jazan 45142, Saudi Arabia

**Keywords:** cardiovascular disease, genetic expression, inflammasome, periodontal medicine

## Abstract

**Simple Summary:**

Researchers have been studying the relationship between gum disease and cardiac health for decades. Gum disease develops when a sticky bacterial biofilm known as plaque forms around the teeth. The bacterial plaque and its toxic byproducts sometimes migrate inside the blood vessels and get lodged in the arterial wall known as atheroma which is found to be a leading cause for heart disease. Various host products are seen to be elevated in the human body as the result of the inflammation. We focused on the relationship between the two biomolecules namely NLRP3 (rs4612666) and CARD8 (rs2043211) with gum and heart disease. Seventy patients were selected for the study and were divided into two groups. Group I consisted of patients with gum diseases only and Group 2 consisted of patients with both gum and heart disease. On analysis, certain factors such as age, cholesterol levels and poor oral health were observed to be higher in patients with both gum and heart disease. The dental plaque from the teeth and the blood drawn from these patients were collected for the testing of the above biomolecules. These were found to be higher in patients with both gum and the heart diseases. Thus, the higher levels of the above biomolecules indicated that patients with severe gum disease and poor oral health may develop a risk to develop heart problems in the future. Thus, early prevention of the oral diseases and gum ailments might reduce the risk of the future heart diseases.

**Abstract:**

Background: The existing data show that inflammasomes play a role in periodontal disease pathogenesis. However, their role in the pathogenesis of periodontitis and coronary heart disease remains unclear. This study had the objective of assessing NLRP3 (rs4612666) and CARD8 (rs2043211) gene polymorphisms in dental plaque and blood of generalized chronic periodontitis (CP) patients in the presence and absence of coronary heart disease (CHD). Methods: A total of 70 subjects were divided into two groups, including CP and CP + CHD subjects. Demographic variables, periodontal, and cardiac parameters were recorded from both groups. Subgingival plaque and blood samples were obtained from both groups and were subjected to further molecular analysis for NLRP3 (rs4612666) and CARD8 (rs2043211) expression and allele change using conventional polymerase chain reaction (PCR) and gene sequencing (Sanger’s method). Results: Amongst the demographic variables, age, and monthly income were statistically significant between the two groups. Plaque index (PI), clinical attachment level (CAL), high-density lipoprotein (HDL), and low density-lipoprotein (LDL) exhibited statistically significant levels between the two groups. NLRP3 (rs4612666) and CARD8 (rs2043211) genes showed a statistically significant association of allele change (frequency) among the groups. In general, when all of the parameters were compared to the allele change of the genes, statistically significant relationships were found between the two groups. Conclusions: The present study expressed an allele change of the investigated genes which could profoundly affect the pathobiology of the two diseases under investigation.

## 1. Introduction

Periodontal disease is the most common chronic infectious-inflammatory disease affecting supporting structures of the teeth that manifests as gingivitis and periodontitis. Interaction of the microorganisms with the host determines the course and extent of the resulting disease [1]. Periodontitis is a disease initiated and sustained by microbial dysbiosis. When infection occurs, innate immunity is the first line of defense, and it recognizes the microorganisms, their toxins, and chemical compounds but also causes tissue destruction due to a mechanism termed bystander damage [2].

Inflammasomes have been documented as an important part of the innate immune response that operates in periodontal pathogenesis [1]. Nucleotide-binding oligomerization domain-like receptor (NLR) inflammasomes are intracellular pattern recognition receptors (PRRs) that detect PAMPs [1]. Inflammasomes recognize a wide range of stimuli that include both microbial and self-molecules [1]. Through the recruitment of cysteine proteinase caspase-1, NLRs activate intracellularly stored pro-IL-1β, which is subsequently released from the cell. The activation of NLRP3/ASC/caspase 1 multiprotein complex, also known as the NLRP3 inflammasome, has recently been shown to be one of the components of innate immune responses that may impact the activity of chronic periodontitis. In response to microbiological, physical, and chemical stimuli, these multiprotein complexes develop. Cell stressors, bacteria, and viruses can also activate NLRP3 [2].

NLRP3 is an inflammasome that activates caspase 1 and secretes interleukin-1β (IL-1β) and interleukin-18 (IL-18). NLRP3 detects PAMPs such as lipopolysaccharides (LPS), which is an important component of the cell wall of Gram-negative microbes which also function as the primary perpetrators of the host’s immune response and other PAMPs including muramyl dipeptide (MDP), bacterial ribonucleic acid (RNA), viral RNA, and deoxyribonucleic acid (DNA) [3]. Caspase recruitment domain (CARD) binds to NLRP during inflammasome activation and forms the assembly of the NLRP inflammasome complex. Caspase activity during cell death is regulated by CARD-containing proteins such as CARD8, which is also a binding partner of NLRP3 [4].

Coronary heart disease has been recognized as a major systemic inflammatory component that emphasizes similarities with periodontal diseases. Linking mechanisms between periodontal disease and coronary heart disease include shared risk factors, increased fibrinogen, the role of white blood cell (WBC), the effect of bacterial LPS, bacteria, and the role of C-reactive protein (CRP), which acts as a strong biomarker [5]. Increased systemic biomarkers of inflammation associated with periodontal disease have been interpreted as a mechanistic link between oral infections and cardiovascular diseases.

Variants were acquired over the lifetime of an organism, whereas polymorphism occurring in genetic susceptibility is the increased likelihood or chance of developing a particular disease due to the presence of one or more gene mutations and/or a family history that indicates an increased risk of the disease. A haplotype is a group of genes inherited together because of genetic linkage. Epigenetics is the study of how behaviors and environment can cause changes that affect the functioning of genes. A mutation is a “DNA a population when the observed variation from individual to individual is not maintained by recurrent mutation.” Therefore, based on the above concepts of clinical genomics, the importance of NLRP3 inflammasome and CARD8 gene as potential biomarkers will explain that these genes are highly specific and conserved in inflammatory diseases such as periodontitis and coronary heart disease [6]. Although studies have been done to identify these genetic changes individually in each disease, none of the studies have explored NLRP3 and CARD8 polymorphisms in subjects with the periodontal disease having coronary heart disease.

We hypothesize that periodontitis may exhibit the expression of NLRP3 and CARD8 polymorphisms in subjects with chronic periodontitis having coronary heart disease. The goal of this study was to find NLRP3 (rs4612666) and CARD8 (rs2043211) gene polymorphisms in subgingival plaque samples and blood samples from generalized chronic periodontitis patients with coronary heart disease to better understand the role of these genes in chronic periodontitis and coronary heart disease.

## 2. Materials and Methods

### 2.1. Study Design

**Figure 1:**
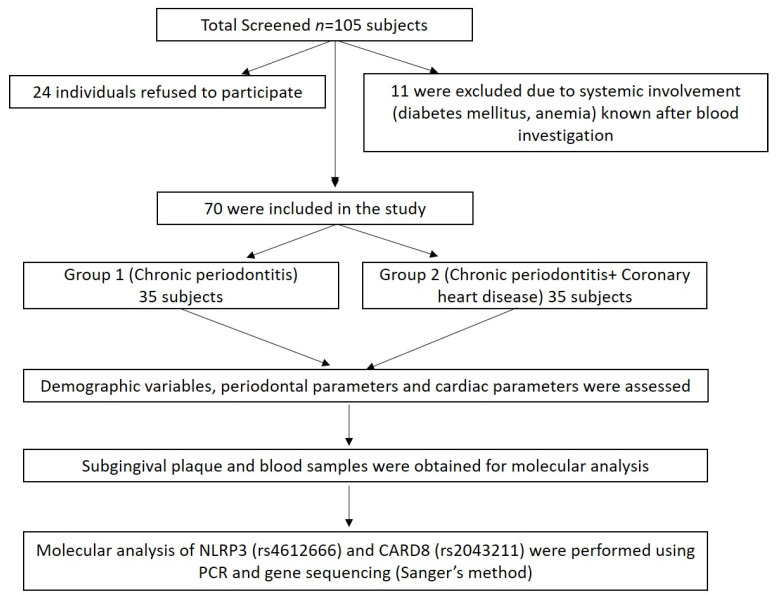
Summary of the study design.

A total of 105 subjects (males) aged 30–65 years from the Department of Periodontics, Meenakshi Ammal Dental College and Hospital, Chennai, and Frontier Lifeline Hospital, Mogappair, Chennai were screened for the study during the period, December 2018 to November 2020. Of these, 24 individuals refused to participate in the study, and 11 were excluded due to systemic disease involvement such as diabetes mellitus and anemic conditions known after blood investigation (Figure 1).

Finally, 70 subjects were divided into two groups: Group I comprised of chronic periodontitis subjects without coronary heart disease (CP *n* = 35) and Group II comprised of chronic periodontitis with coronary heart disease (CP + CHD *n* = 35) (Figure 1). The power analysis of the study was calculated to be 80% with a minimum sample size of 70 samples based on the prevalence of periodontitis and coronary heart disease. The study was approved by the “Institutional Review Board”, MAHER-Deemed to be University, Chennai (MADC/IRB-XXV/2018/391) and registered under clinicaltrials.gov with ID No. NCT04127487, and the study was conducted following the Helsinki Declaration of 1975, as revised in 2013. The study protocol was explained to all the subjects and written informed consent was obtained.

### 2.2. Inclusion and Exclusion Criteria

Inclusion criteria for the selected subjects were as follows: (1) Patients who readily consented to participate; (2) Patients in the range of 30–65 years (male); (3) at least 20 remaining natural teeth; (4) subjects with CP only group, individuals with periodontitis satisfying the case definition by a World Workshop in Classification of Periodontal Diseases and Conditions 1999, were recruited; (5) subjects with CP + CHD group, and coronary heart disease was confirmed by the cardiologist.

Exclusion criteria for both the study groups were as follows: (1) Systemic conditions such as renal disease, type I and type II diabetes mellitus, respiratory diseases, advanced malignancies, liver disease, rheumatoid arthritis, allergy, and HIV infection; (2) Female patients were excluded due to hormonal variations; (3) use of corticosteroids, antibiotics within the previous three months of investigation; (4) Smokers and individuals who quit smoking less than six months ago; (5) periodontal therapy within the preceding six months.

### 2.3. Demographic, Clinical, and Cardiac Parameters

All the participants underwent documentation of their demographic variables such as age, height, weight, BMI, waist–hip ratio, and monthly income; periodontal parameters such as plaque index (PI) [7], bleeding on probing (BOP) [8], probing pocket depth (PPD), clinical attachment level (CAL) and cardiac parameters such as total cholesterol levels (TC), High-density lipoprotein levels (HDL), Low-density lipoprotein levels (LDL), total triglyceride levels (TG), Systolic blood pressure (SBP), and Diastolic blood pressure (DBP) were recorded. Periodontal parameters were recorded using a Manual Williams periodontal probe, (Hu-Friedy, Chicago, IL, USA) to the nearest millimeter from six sites per tooth.

### 2.4. Subgingival Plaque and Blood Samples Collection

After periodontal diagnosis, subgingival dental plaque samples were obtained from the deepest periodontal pocket from all participants with the help of universal curettes† (Manual Universal Curettes, Hu-Friedy) and were transferred to the sterilized vial containing 95% absolute ethanol in the two groups and stored at −80 °C.

For the collection of blood samples, 2 mL of venous blood were collected from the antecubital vein by venipuncture using a 20-gauge needle with a 2 mL syringe from each participant in both groups. The collected blood sample was immediately transferred to EDTA vacutainer and stored at 4 °C for further analysis.

### 2.5. PCR Analysis

Furthermore, 100 µL of DNA were isolated from subgingival plaque samples using Xpress DNA kit ‡ (Xpress DNA kit, MagGenome Technologies Pvt. Ltd., Cochin, India) and blood samples using QIAamp DNA mini kit § (QIAamp1 DNA mini kit, Qiagen Sciences, Germantown, MD, USA), respectively, as per the manufacturer’s protocol. After isolation of DNA, quantification was done using nanodrop machine ‖ (NANODROP, Thermo Fisher Scientific, Wilmington, DE, USA) and PCR ¶ (PCR, Agilent Technologies, SureCycler 8000, Santa Clara, CA, USA) analysis was processed. The reaction mix (20 μL) was prepared by adding 10 μL of PCR master mix solution ** (Red dye PCR Mastermix, Ampliqon, Odense, Denmark), 2 μL forward primer #, 2 µL reverse primer # (Oligo nucleotide primer, PrimeX Oligo Sythesis and Purification Services, Gujarat, India), 2.5 µL DNA, and the solution was made up to 20 µL with sterile water. The standardization of the protocol was achieved by using 2.5 µL of DNA for all the samples being analyzed. 

The reaction conditions for both the primers were as follows: Initial denaturation at 95 °C for 10 min, followed by 40 cycles of PCR with denaturation at 95 °C for 15 s, annealing at 55 °C for 60 s and extension at 72 °C for 60 s. The final extension was done at 72 °C for 60 s.

### 2.6. Agarose Gel Electrophoresis

**Figure 2:**
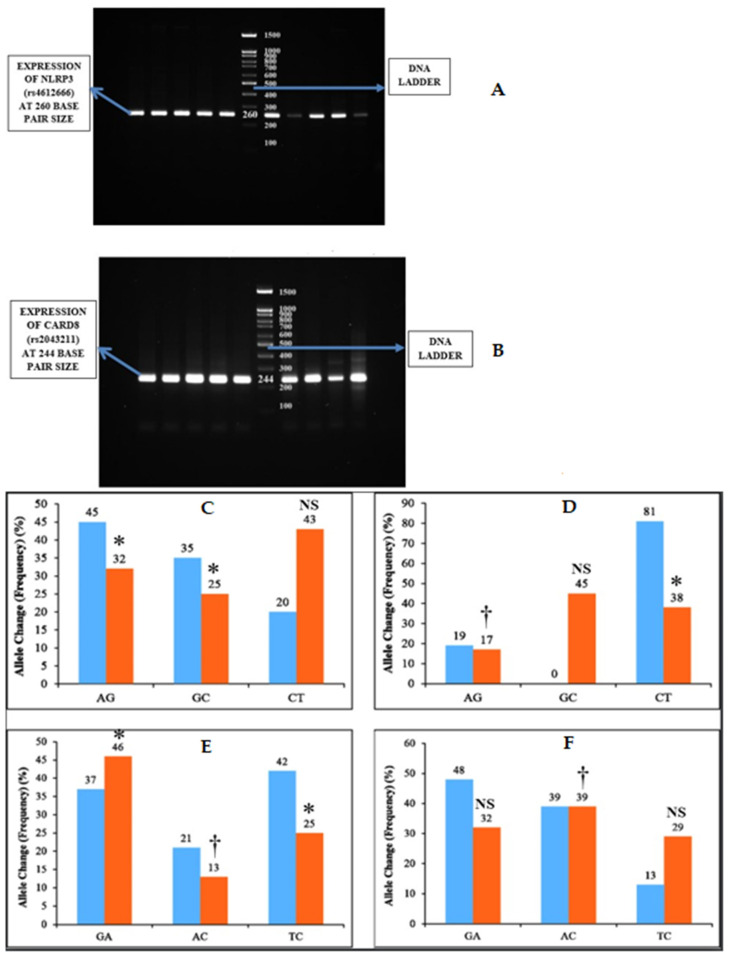
Inflammasome expression and allele change among the two groups. (**A**,**B**) NLRP3 (rs4612666) was expressed as PCR bands at 260 bp size and CARD8 (rs2043211) as PCR bands at 244 bp size. (**C**) represents the Allele change of NLRP3 (rs4612666) in subgingival plaque and (**D**) represents the allele change of NLRP3 (rs4612666) in blood samples among the two groups. (**E**) represents allele change of CARD8 (rs2043211) in subgingival plaque and (**F**) represents allele change of CARD8 (rs2043211) in blood samples among the two groups. The horizontal lane in (**A**) represents the expression of NLRP3 and (**B**) represents CARD8 polymorphism. * *p* < 0.05 is considered to be statistically significant, † *p* < 0.01 is considered to be highly statistically significant, NS—Non-significant.

One gram of agarose was dissolved in 50 mL of Tris-acetate EDTA 1× (TAE) buffer †† (10× TAE Buffer, Sisco Research Laboratories Pvt. Ltd, Mumbai, India) and heated. Then, the agarose gel was cast with 5 µL of ethidium bromide. The solution was then poured into the gel cassettes and allowed to cool and solidify. Then, the gel cassette was placed in the tank containing 50× TAE buffer. Five microliters of PCR product were then loaded to each well with a 100 bp DNA ladder ‡‡ (DNA Ladder 1kb, Gene Direx, Taoyuan, Taiwan) to track the molecular weight of the PCR product. Then, the electrophoresis was carried out at 150 mA, and the gels were visualized in Gel Doc machine (Figure 2).

### 2.7. Gene Sequencing

In Sanger sequencing, a DNA primer was used as a starting point for DNA synthesis and complementary template DNA (the DNA to be sequenced) is produced. Deoxynucleotide triphosphates (dNTPs: A, G, C, and T) were present in the DNA sequences and the polymerase extends the primer by adding the complementary dNTP to the template DNA strand. The ends of the fragments were then labeled with dyes that indicated their final nucleotide.

### 2.8. Statistical Analysis

Statistical analysis was performed in standard statistical software Statistical Package for Social Sciences (SPSS) version 20.1, Chicago, USA, Inc. Mean and standard deviation were estimated. Data were expressed as mean ± standard deviation.

Comparison of the data was analyzed using one-way analysis of variance (ANOVA), sample *t*-test, and chi-square test, and *p* < 0.05 was considered as the level of significance.

## 3. Results

### 3.1. Demographic Variables, Periodontal and Cardiac Parameters

Results of bivariate comparison among demographic variables and clinical parameters, mean age (*p* < 0.001), monthly income (*p* = 0.034), plaque index (*p* < 0.001), clinical attachment level (*p* < 0.001), high-density lipoprotein (*p* = 0.019), low-density lipoprotein (*p* = 0.006) showed statistically significant levels between the two groups (Table 1). Other demographic variables (height, weight, BMI, waist–hip ratio), periodontal (BOP, PPD), and cardiac parameters (TC, TG, SBP, DBP) did not show any statistical difference (Table 1).

### 3.2. PCR Analysis

The n (%) of (rs4612666) and CARD8 (rs2043211) genes in dental plaque and blood samples of the CP + CHD group were found to be higher than the CP group (Table 2). However, both of the inflammasomes were not statistically significant in subgingival plaque samples (NLRP3 *p* = 0.177) (CARD8 *p* = 1.000), but, in blood samples, it showed a significant difference (*p* = 0.000) (Table 2).

### 3.3. Gene Sequencing Analysis

The NLRP3 (rs4612666) gene showed statistically significant association of AG (*p* = 0.021), GC (*p* = 0.004) allele change (frequency) in subgingival plaque samples, and AG (*p* = 0.01), CT (*p* = 0.0052) allele change (frequency) in blood samples among both of the groups (Table 3) (Figure 2).

Similarly, the CARD8 (rs2043211) gene showed statistically significant association of GA (*p* = 0.052), AC (*p* = 0.01), and TC (*p* = 0.032) allele change (frequency) in subgingival plaque samples and AC (*p* = 0.01) allele change (frequency) in blood samples among both of the groups (Table 4) (Figure 2).

Generally, upon comparison of demographic, periodontal, and cardiac parameters with the allele change (frequency) of NLRP3 (rs4612666) in subgingival plaque samples showed statistically significant association with age (*p* = 0.01), PI (*p* = 0.05), BOP (*p* = 0.05), CAL (*p* = 0.01), and LDL (*p* = 0.05). NLRP3 (rs4612666) in blood samples also showed statistically significant association with age (*p* = 0.01), PI (*p* = 0.05), PPD (*p* = 0.05), and CAL (*p* = 0.01) (Table S1).

Similarly, a comparison of demographic, periodontal, and cardiac parameters with the allele change (frequency) of CARD8 (rs2043211) in subgingival plaque samples showed statistically significant association with age (*p* = 0.001), PI (*p* = 0.001), PPD (*p* = 0.001), CAL (*p* = 0.001), and diastolic blood pressure (*p* = 0.05). CARD8 (rs2043211) in blood samples also showed statistically significant association with height (*p* = 0.05), BMI (*p* = 0.01), PPD (*p* = 0.01), CAL (*p* = 0.01), and systolic blood pressure (*p* = 0.05) (Table S2).

## 4. Discussion

The oral cavity comprises a varied ecosystem that harbors an immense number of microorganisms. It acts as a reservoir for the systemic dissemination of bacteria. In chronic periodontitis, inflammatory response turns out to be chronic when pathogenic bacteria continuously circulate that cannot be controlled by the acute immune response, thereby resulting in uncertain inflammation, obliteration of alveolar bone and soft tissue, and fibrosis [9].

The destruction of gingival epithelium by periodontal pathogens results in the production of local inflammatory mediators from the periodontal pocket that reaches the systemic circulation, thus enabling immune cell recruitment. In addition, bacteria can either indirectly or directly circulate in the bloodstream [10]. Therefore, under conditions where there is a disposition (e.g., genetic, lifestyle) toward cardiovascular disease (CVD), the bacterial components and systemic inflammatory mediators can potentially accelerate plaque formation. To this point, periodontal pathogens have been identified in various tissues and organs, particularly in the cardiovascular system [11,12].

Furthermore, several research studies have revealed new information on inflammasome-related proteins across the periodontal disease spectrum, and persuasive evidence suggests that oral biofilms may influence the production of inflammasomes and their associated cytokine targets at the same time. Inflammasomes act as chief regulators of the innate immune system in chronic inflammatory diseases by defending microbial pathogens, thereby controlling and reducing the invading microbes. They play an important role in the progression of periodontal disease and inflammasome-associated inflammatory mediators. Several types of NLRs (NLRP1, NLRP2, NLRP3, NLRP6, NLRP12, etc.) are found to be associated with systemic diseases [1]—out of which, NLRP3 and CARD8 inflammasome have been implicated in the pathogenesis of several inflammatory diseases like atherosclerosis, Crohn’s disease, gout, and periodontal disease [1].

The NLRP3 inflammasome is activated in response to the array of stimuli, leading to the theory that the dissimilar agonists induce similar downstream events that are sensed by NLRP3. NLRP3 activation leads to conversion of inactive caspase-1 to active caspase-1 [4]. On the other hand, CARD8 inflammasome is a known member of the CARD family. It is a co-regulator in apoptotic signaling pathways and inflammatory pathways. CARD8 is involved in subduing NF-kB activation, which in turn suppresses the immune responses [13]. During cell death, CARD8 binds with NLRP3 and regulates caspase activity. CARD8 also induces pyroptosis by Val-boroPro in human T cells [14].

The vast majority of the biology of a newly sequenced genome is inferred from the set of encoded proteins. Predicting this set is therefore invariably the first step after the completion of the genome DNA sequence. Protein coding sequences are DNA sequences that are transcribed into mRNA and in which the corresponding mRNA molecules are translated into a polypeptide chain. Literature suggested that genetic contributions (identification of the protein-coding sequences) to inflammatory mediated diseases such as chronic periodontitis and coronary heart disease are important and sometimes perplexing due to factors such as genetic heterogeneity, gene-environmental interactions, and incomplete penetrance [15]. This study is the first explorative approach evaluating NLRP3 (rs4612666) and CARD8 (rs2043211) polymorphisms in dental plaque and blood of periodontitis patients with coronary heart disease.

The demographic variables such as height, weight, BMI, and the waist–hip ratio did not show any statistical difference, whereas age and monthly income showed statistically significant levels between the two groups (Table 1). The age was, however, found to be significantly higher in CP + CHD (56.45 ± 8.32) than CP group (43.42 ± 9.08) with a *p* < 0.001, which shows that age is one of the confounding factors for periodontal and coronary heart disease. Literature proposed that previous reports of the National Heart, Lung, and Blood Institute showed the prevalence of men for coronary heart disease increases at 45 years and above [16]. Henceforth, in the present study, coronary heart disease subjects with periodontitis showed a higher mean age (˃40 years). The mean monthly income in subjects with CP + CHD was comparatively lower than the CP group, which was statistically significant (Table 1). The existing results are following a study that demonstrated that lower or middle social classes presented a greater risk for coronary heart disease than higher social class individuals [17].

Periodontal parameters such as PI and CAL showed statistically significant higher levels in CP + CHD (PI 0.95 ± 0.15) (CAL 5.69 ± 0.55) than the CP (PI 0.60 ± 0.12) (CAL 4.48 ± 0.60) group with a *p* < 0.001, respectively suggesting severity of periodontitis in CP + CHD subjects. Even though periodontal parameters measured clinically cannot be a sole indicator of disease severity and its association with CHD remains inconclusive, the link proven from various studies reports a causal relationship. A similar study stated that individuals with a plaque index of ≥65% were positively associated with periodontal destruction, which correlated with the findings of the current study [18]. The correlation between clinical attachment level and cardiovascular diseases was also explored in the previous literature [19,20]. Moreover, epidemiological data [21,22] revealed an association of periodontal parameters such as bleeding on probing, pocket depth, and clinical attachment level with the degree and number of obstructed coronary arteries, which indicated a positive correlation for periodontal and cardiovascular disease [23]. Thus, in this study, the higher values of periodontal parameters established a strong link between clinical parameters of periodontitis and CHD.

Likewise, cardiac parameters such as TC, TG, SBP, and DBP showed a higher mean in CP + CHD than the CP group but were not statistically significant, whereas HDL and LDL revealed statistically significant mean levels in CP + CHD (HDL 36.37 ± 8.28) (LDL 129.11 ± 39.50) than CP subjects (HDL 40.60 ± 6.26) (LDL 107.31 ± 23.16), with a *p* = 0.019 and 0.006, respectively. This is following the study, where the mean levels of HDL, LDL, and TC were significantly higher in coronary heart disease patients with and without periodontitis, except for TG, which was found to be statistically nonsignificant [24].

In periodontitis conditions, as the antioxidant levels are decreased, there are chances of increased oxidation of LDL and decreased HDL, thereby increasing the oxidative state. In oxidative stress, HDL proteins undergo alterations and diminish the atheroprotective properties of HDL, thereby influencing artherogenesis [25]. Understanding the facts, that cardiac parameters take part in an inflammatory pathway for periodontal and cardiovascular diseases, a probable systemic inflammation via periodontal pathogenic bacteria may lead to increased LDL and decreased HDL levels in the bloodstream, thus suggesting an increased risk of coronary heart disease.

The genetic information expressed within the human genome is capable of controlling the production of inflammatory mediators and also acts as a potential therapeutic target for periodontal and cardiovascular diseases. Molecular genetic research identifies individual genetic background that contributes to the development of both periodontal and coronary heart diseases. In addition, inflammation-associated genetic variance has been anticipated to increase susceptibility to periodontitis and coronary heart diseases, but the association was not confirmed. Thus, we decided to investigate the role of NLRP3 and CARD8 SNPs in expressing inflammatory proteins. To the best of our understanding, no study so far has explored the involvement of NLRP3 and CARD8 single nucleotide polymorphisms (SNPs) in chronic periodontitis subjects with and without coronary heart disease.

The foremost findings reported significantly higher mean levels of NLRP3 (rs4612666) and CARD8 (rs2043211) polymorphism in plaque and blood of CP + CHD than the CP group. However, this was not statistically significant in subgingival plaque samples (NLRP3 *p* = 0.177) (CARD8 *p* = 1.000) but was found to be significant in blood samples (*p* = 0.000) (Table 2). Samples were selected for sequencing-based upon the PCR band strength and intensity, taking into account the combination of the polymorphisms Q705K (rs35829419) and C10X (rs2043211) in the NLRP3 and CARD8 genes shown to be expressed in cardiovascular patients [26]. Significant deviation from the Hardy–Weinberg equilibrium X^2^ (HWE) denotes genotyping error and sampling bias. After examining both NLRP3 and CARD8 SNPs in plaque and blood in the current investigation, it was discovered that both genetic variations were in proportions compatible with HWE X2 among CP and CP +CHD participants, implying that the allele frequencies of these SNPs in the population are stable.

In the present study, allele change AG of NLRP3 (rs4612666) in subgingival plaque and blood samples showed a significant odds ratio of 1.07 and 1.06 among CP and CP + CHD group, respectively. Similarly, allele GC showed a significant odds ratio of 0.93 in subgingival plaque samples and a nonsignificant odds ratio of 0.89 in blood samples. In addition, allele CT obtained a statistically nonsignificant odds ratio of 0.98 in subgingival plaque samples and statistically significant odds of 0.95 in blood samples (Table 3), while former studies have shown an odds ratio of 3.95 of CT allele when comparing periodontally diseases subjects with the healthy group [27]. A recent study observed a nonsignificant odds ratio of 0.464 of CT allele change in positive microembolic signals atherosclerotic patients when compared with negative microembolic signals atherosclerotic patients [28]. The documentation of allele changes of AG and GC about the NLRP3 gene is considered a novel component of the study as it has not been previously researched.

On the other hand, for CARD8 (rs2043211) gene polymorphism, GA, AC, and TC alleles showed a significant odds ratio (GA 1.14), (AC 0.92), and (TC 0.91) in subgingival plaque and blood (AC 0.99) samples, respectively. Conversely, in the present study, there was no statistically significant odds ratio of (GA 1.2) and (TC 0.93) in blood samples (Table 4). According to a previous study, there is a substantial connection in AT allele frequency between arteriosclerosis obliterans and healthy male controls, with an odds ratio of 1.297 [29]. The documentation of allele changes of GA, AC, and TC in the CARD8 gene is also a novel finding of the present study, which has not been previously documented.

In concordance with previous investigations, those individuals carrying the rare variant of the NLRP3 (rs4612666) and CARD8 (rs2043211) polymorphism would therefore have an increased inflammatory activity. It is also important to highlight that a positive association of allele change of NLRP3 (rs4612666) with age, PI, BOP, CAL, and LDL were obtained in subgingival plaque samples. However, in blood samples, significant associations with age, PI, PPD, and CAL were seen (Table S1). Likewise, for CARD8 (rs2043211), the significant association of age, PI, PPD, CAL, and DBP were observed in subgingival plaque samples. Similarly, in blood samples, a statistically significant association with height, BMI, PPD, CAL, and SBP were identified (Table S1). A recent study also showed a stronger association of the extent of periodontitis with an allele frequency of NLRP3 (rs4612666) between periodontitis subjects and healthy controls [27].

In the current study, however, no significant differences in a few clinical parameters were found between genotype groups, indicating that anatomic differences, local irritants, and pathogens may mask the genetic influence of NLRP3 and CARD8 SNPs on the development of chronic periodontitis and coronary heart disease. However, conflicting evidence has also been published previously regarding the rs4612666 and rs2043211polymorphism and several different diseases with inflammatory background, like inflammatory bowel disease, increased severity of rheumatoid arthritis, increased risk of Alzheimer’s disease, and other inflammatory diseases.

SNPs are considered as genetic markers present in the human genome, where specific SNPs are responsible for specific diseases that provide significant transformations and associations between various diseases. Several SNPs have been estimated rendering the role of different genes in the pathogenesis of periodontal and coronary artery disease. Thus, these allele changes or frequency in SNPs have made it possible for early detection of various diseases like periodontal disease, diabetes mellitus, osteoporosis, coronary artery disease, etc. Despite the controversy surrounding genetic association research, the study of SNPs’ supposed functionality in the oral environment may reveal their harmful significance in inflammatory disorders including periodontitis and coronary heart disease.

Scarce research has been conducted on the association between inflammasome SNPs in periodontitis and coronary heart disease. To the authors’ knowledge, this is the first study to systematically analyze this relationship. However, some limitations need to be addressed in future investigations related to this study. First, longitudinal studies are required to explore the role of NLRP3 (rs4612666) and CARD8 (rs2043211) as a therapeutic marker for both periodontitis and coronary heart disease. Second, only the diagnosis is made and the severity of coronary heart disease was not evaluated. Third, larger sample sizes with less dropout of samples are required for future longitudinal studies. Thus, further studies are required to evaluate and explore the prognostic and therapeutic implications for inflammasome activation in the progression of periodontal and coronary heart disease.

## 5. Conclusions

Owing to these facts in the future, these results could form a basis for various genetic mutation research studies concerning inflammatory diseases, exploring the association of periodontitis and coronary heart disease. SNPs predict strong or weak correlations to one of these several comorbid diseases. Increased understanding of the gene polymorphisms may lead to new approaches in the development of novel therapeutics for periodontal disease, atherosclerosis, and other inflammatory diseases. Thus, early detection of SNPs can be a greater asset for the detection of periodontitis and coronary artery patients foreseeing its stronger or weaker associations and helps in obtaining its correlations with its comorbid diseases.

## Figures and Tables

**Table 1:** Comparison of demographic variables, periodontal parameters, and cardiac parameters between Group I and Group II.

Variable	Group I Mean ± Standard Deviation	Group II Mean ± Standard Deviation	*p*-Value
Age (years)	43.42 ± 9.08	56.45 ± 8.32	<0.001 ^‡^
Height (cm)	166.00 ± 6.11	164.08 ± 9.43	0.317 ^NS^
Weight (Kg)	65.40 ± 6.40	67.50 ± 10.43	0.312 ^NS^
Body Mass Index (BMI)	23.65 ± 2.49	24.84 ± 3.14	0.083^NS^
Waist Hip ratio	0.83 ± 0.04	0.84 ± 0.04	0.474 ^NS^
Monthly Income (Rs)	23,342.86 ± 7870.05	22,000 ± 7352.47	0.034 *
Plaque Index	0.60 ± 0.12	0.95 ± 0.15	<0.001 ^‡^
Bleeding on Probing (%)	71.22 ± 8.09	76.76 ± 24.31	0.205 ^NS^
Probing Pocket Depth (mm)	3.71 ± 0.677	18.03 ± 79.51	0.291 ^NS^
Clinical Attachment Level (mm)	4.48 ± 0.60	5.69 ± 0.55	<0.001 ^‡^
Total Cholesterol (mg/dL)	173.14 ± 28.91	187.51 ± 46.47	0.125 ^NS^
High-Density Lipoprotein (mg/dL)	40.60 ± 6.26	36.37 ± 8.28	0.019 ^†^
Low-Density Lipoprotein (mg/dL)	107.31 ± 23.16	129.11 ± 39.50	0.006 ^†^
Triglycerides (mg/dL)	133.11 ± 29.98	144.40 ± 55.94	0.297 ^NS^
Systolic Blood Pressure (mm Hg)	125.14 ± 8.86	125.42 ± 9.50	0.897 ^NS^
Diastolic Blood Pressure (mm Hg)	78.28 ± 5.13	80.28 ± 6.17	0.145 ^NS^

* *p* < 0.05 is considered to be statistically significant; † *p* < 0.01 is considered to be highly statistically significant; ‡ *p* < 0.001 is considered to be very highly statistically significant; NS–Non-significant.

**Table 2:** Comparison of NLRP3 (rs4612666) and CARD8 (rs2043211) gene expression between Group I and Group II.

Gene Expression	Group 1	Group 2	*p*-Value
	n (%)	n (%)	
NLRP3 (rs4612666) (Subgingival plaque sample)	82.9	88.6	0.177 ^NS^
NLRP3 (rs4612666) (Blood sample)	71.4	100	<0.001 ^‡^
CARD8 (rs2043211) (Subgingival plaque sample)	80	80	1.000 ^NS^
CARD8 (rs2043211) (Blood sample)	68.6	97.1	<0.001 ^‡^
NLRP3 (rs35829419) (Subgingival plaque sample)	0	5.70	0.003 ^‡^
NLRP3 (rs35829419) (Blood sample)	11.43	34.29	<0.001 ^‡^
IL-1β (+3954) (Subgingival plaque sample)	82.9	91.4	0.032 ^†^
IL-1β (+3954) (Blood sample)	71.4	100	<0.001 ^‡^

† *p* < 0.01 is considered to be highly statistically significant; ‡ *p* < 0.001 is considered to be very highly statistically significant; NS–Non significant.

**Table 3:** Allele change (Frequency) of NLRP3 (rs4612666) gene polymorphisms among Group I and Group II in subgingival plaque and blood samples.

**NLRP3 (rs4612666) Subgingival Plaque**	**Group I**	**Group II**	**Odds Ratio ^b^** **(Class Interval)**	***p*** **-Value ^c^**
***n***	20	28		
**Frequency%**	57.1%	80.0%		
**Allele change (Frequency) ^a^ n (%)**				
**AG**	9 (45%)	9 (32%)	1.07 (1.02–1.20)	0.021 *
**GC**	7 (35%)	7 (25%)	0.93 (0.87–1.16)	0.04 *
**CT**	4 (20%)	12 (43%)	0.98 (0.91–1.15)	0.78 ^NS^
**X^2^**	28.1	47.8		
***p*** **-value ^c^**	0.05 *	0.001 ^‡^		
**Allele Expression**				
**A**	16 (40%)	16 (29%)		
**C**	11 (28%)	19 (34%)		
**G**	9 (22%)	9 (16%)		
**T**	4 (10%)	12 (21%)		
**NLRP3 (rs4612666) Blood**	**Group I**	**Group II**	**Odds Ratio ^b^** **(Class Interval)**	***p*** **-Value ^c^**
***n***	16	29		
**Frequency%**	45.7%	82.9%		
**Allele change (Frequency) ^a^ n (%)**				
**AG**	3 (19%)	5 (17%)	1.06 (1.02–1.25)	0.01 ^†^
**GC**	0	13 (45%)	0.89 (0.83–1.02)	0.82 ^NS^
**CT**	13 (81%)	11 (38%)	0.95 (0.85–1.1)	0.052 *
**X^2^**	48	48		
***p*** **value ^c^**	0.62 ^NS^	0.047 *		

* *p* < 0.05 is considered to be statistically significant; † *p* < 0.01 is considered to be highly statistically significant, ‡ *p* < 0.001 is considered to be very highly statistically significant. NS–Non significant; ^a^ Values are given as n (%) of individuals ^b^ Odds ratio (95% confidence interval) ^c^ Two-sided Pearson’s Chi-square test (X^2^); A–Adenine, C–Cytosine, G–Guanine, T–Thymine.

**Table 4:** Allele change (Frequency) of CARD8 (rs2043211) gene polymorphisms among Group I and Group II in subgingival plaque and blood samples.

**CARD8 (rs2043211) Subgingival Plaque**	**Group I**	**Group II**	**Odds Ratio ^b^** **(Class Interval)**	***p*** **-Value ^c^**
***n***	19	24		
**Frequency%**	54.3%	68.6%		
**Allele change (Frequency) ^a^ n (%)**				
**GA**	7 (37%)	11 (46%)	1.14 (1.04–1.19)	0.052 *
**AC**	4 (21%)	3 (13%)	0.92 (0.88–1.09)	0.01 ^†^
**TC**	8 (42%)	10 (25%)	0.91 (0.87–1.07)	0.032 *
**X^2^**	35.1	35.1		
***p*** **-Value** **^c^**	0.05 *	0.001 ^‡^		
**Allele Expression**				
**A**	11 (29%)	14 (29%)		
**C**	12 (32%)	13 (27%)		
**G**	7 (18%)	11 (23%)		
**T**	8 (21%)	10 (21%)		
**CARD8 (rs2043211) Blood**	**Group I**	**Group II**	**Odds Ratio ^b^** **(Class Interval)**	***p*** **-Value ^c^**
***n***	23	28		
**Frequency%**	65.7%	80.0%		
**Allele change (Frequency) ^a^ n (%)**				
**GA**	11 (48%)	9 (32%)	1.2 (1–1.30)	0.496 ^NS^
**AC**	9 (39%)	11 (39%)	0.99 (0.85–1.05)	0.01 ^†^
**TC**	3 (13%)	8 (29%)	0.93 (0.90–1)	0.78 ^NS^
**X^2^**	29.3	38.6		
***p*** **-Value** **^c^**	0.073 ^NS^	0.05 *		

* *p* < 0.05 is considered to be statistically significant; † *p* < 0.01 is considered to be highly statistically significant; ‡ *p* < 0.001 is considered to be very highly statistically significant. NS–Non significant; ^a^ Values are given as n (%) of individuals; ^b^ Odds ratio (95% confidence interval); ^c^ Two-sided Pearson’s Chi-square test (X^2^).; A–Adenine, C–Cytosine, G–Guanine, T–Thymine.

## Data Availability

Not applicable.

## Supplementary Materials

The following are available online at https://www.mdpi.com/article/10.3390/biology10070592/s1, Table S1: Overall comparison of the demographic variables, periodontal parameters and cardiac parameters with the allele change (frequency) of the NLRP3 (rs4612666) in subgingival plaque and blood samples for both the groups; Table S2: Overall comparison of the demographic variables, periodontal parameters and cardiac parameters with the allele change (frequency) of the CARD8 (rs2043211) in subgingival plaque and blood samples for both the groups.

## References

[1] Lamkanfi M., Dixit V.M. (2012). Inflammasomes and their roles in health and disease. Annu. Rev. Cell Dev. Biol..

[2] Belibasakis G.N., Guggenheim B., Bostanci N. (2013). Down-regulation of NLRP3 inflammasome in gingival fibroblasts by subgingival biofilms: Involvement of *Porphyromonas gingivalis*. Innate Immun..

[3] Chen G., Shaw M.H., Kim Y.-G., Nuñez G. (2009). NOD-like receptors: Role in innate immunity and inflammatory disease. Annu. Rev. Pathol..

[4] Broz P., Dixit V.M. (2016). Inflammasomes: Mechanism of assembly, regulation and signalling. Nat. Rev. Immunol..

[5] Li X., Kolltveit K.M., Tronstad L., Olsen I. (2000). Systemic diseases caused by oral infection. Clin. Microbiol. Rev..

[6] Paramel G.V., Sirsjö A., Fransén K. (2015). Role of genetic alterations in the NLRP3 and CARD8 genes in health and disease. Mediat. Inflamm..

[7] Silness J., Loe H. (1964). Periodontal disease in pregnancy. Ii. Correlation between oral hygiene and periodontal condtion. Acta Odontol. Scand..

[8] Ainamo J., Bay I. (1975). Problems and proposals for recording gingivitis and plaque. Int. Dent. J..

[9] (2010). cochrane New Cochrane Systematic Reviews—Cochrane Oral Health Group. J. Evid. Based Dent. Pract..

[10] Lockhart P.B., Brennan M.T., Thornhill M., Michalowicz B.S., Noll J., Bahrani-Mougeot F.K., Sasser H.C. (2009). Poor oral hygiene as a risk factor for infective endocarditis-related bacteremia. J. Am. Dent. Assoc..

[11] Pessi T., Karhunen V., Karjalainen P.P., Ylitalo A., Airaksinen J.K., Niemi M., Pietila M., Lounatmaa K., Haapaniemi T., Lehtimäki T. (2013). Bacterial signatures in thrombus aspirates of patients with myocardial infarction. Circulation.

[12] Moreno S., Parra B., Botero J.E., Moreno F., Vásquez D., Fernández H., Alba S., Gallego S., Castillo G., Contreras A. (2017). Periodontal microbiota and microorganisms isolated from heart valves in patients undergoing valve replacement surgery in a clinic in Cali, Colombia. Biomedica.

[13] Bouchier-Hayes L., Martin S.J. (2002). CARD games in apoptosis and immunity. EMBO Rep..

[14] Linder A., Bauernfried S., Cheng Y., Albanese M., Jung C., Keppler O.T., Hornung V. (2020). CARD8 inflammasome activation triggers pyroptosis in human T cells. EMBO J..

[15] Holla L.I., Fassmann A., Muzík J., Vanek J., Vasku A. (2006). Functional polymorphisms in the matrix metalloproteinase-9 gene in relation to severity of chronic periodontitis. J. Periodontol..

[16] Hajar R. (2017). Risk Factors for Coronary Artery Disease: Historical Perspectives. Heart Views.

[17] Janati A., Matlabi H., Allahverdipour H., Gholizadeh M., Abdollahi L. (2011). Socioeconomic status and coronary heart disease. Health Promot. Perspect..

[18] De Macêdo T.C.N., Costa M.d.C.N., Gomes-Filho I.S., Vianna M.I.P., Santos C.T. (2006). Factors related to periodontal disease in a rural population. Braz. Oral Res..

[19] Pejcic A., Kesic L., Brkic Z., Pesic Z., Mirkovic D. (2011). Effect of periodontal treatment on lipoproteins levels in plasma in patients with periodontitis. South Med. J..

[20] Chrysanthakopoulos N.A., Oikonomou A.A. (2017). Periodontal disease as a possible risk factor for atherosclerotic cardiovascular diseases in a Greek adult population. Ann. Res. Hosp..

[21] Humphrey L.L., Fu R., Buckley D.I., Freeman M., Helfand M. (2008). Periodontal disease and coronary heart disease incidence: A systematic review and meta-analysis. J. Gen. Intern. Med..

[22] Tonetti M.S. (2009). Periodontitis and risk for atherosclerosis: An update on intervention trials. J. Clin. Periodontol..

[23] Ketabi M., Meybodi F.R., Asgari M.R. (2016). The association between periodontal disease parameters and severity of atherosclerosis. Dent. Res. J. (Isfahan).

[24] Akkaloori A., Parthasarathi P., Anjum M., Gadde P., Mocherla M., Rao Y. (2014). Association between chronic periodontal disease and cardiovascular risk factor hyperlipidemia. J. Dr. NTR Univ. Health Sci..

[25] Ljunggren S., Bengtsson T., Karlsson H., Starkhammar Johansson C., Palm E., Nayeri F., Ghafouri B., Davies J., Svensäter G., Lönn J. (2019). Modified lipoproteins in periodontitis: A link to cardiovascular disease?. Biosci. Rep..

[26] Varghese G.P., Fransén K., Hurtig-Wennlöf A., Bengtsson T., Jansson J.-H., Sirsjö A. (2013). Q705K variant in NLRP3 gene confers protection against myocardial infarction in female individuals. Biomed. Rep..

[27] Isaza-Guzmán D.M., Medina-Piedrahíta V.M., Gutiérrez-Henao C., Tobón-Arroyave S.I. (2017). Salivary Levels of NLRP3 Inflammasome-Related Proteins as Potential Biomarkers of Periodontal Clinical Status. J. Periodontol..

[28] Cheng L., Yin R., Yang S., Pan X., Ma A. (2018). Rs4612666 Polymorphism of the NLRP3 Gene Is Associated with the Occurrence of Large Artery Atherosclerotic Ischemic Strokes and Microembolic Signals. BioMed Res. Int..

[29] Zhang K., Song W., Li D., Yan J., Chen Y., Qi H., Jin X., Zhao J. (2017). The Association between Polymorphism of CARD8 rs2043211 and Susceptibility to Arteriosclerosis Obliterans in Chinese Han Male Population. Cell. Physiol. Biochem..

