# Supplementary material for: Genetic Polymorphisms of NLRP3 (rs4612666) and CARD8 (rs2043211) in Periodontitis and Cardiovascular Diseases
Source: Biology (Basel). 2021 Jun 27;10(7):592. doi: 10.3390/biology10070592 (PMC8301148; doi:10.3390/biology10070592)
Supplement: Supplementary file 1 [file biology-10-00592-s001.zip › biology-1241977-supplementary.pdf]

biology

Table S1. Overall comparison of the demographic variables, periodontal parameters and cardiac parameters with the allele change (frequency) of the NLRP3 (rs4612666) in subgingival plaque and blood samples for both the groups.

Variable	Allele change (Frequency) Mean ± Standard deviation (Subgingival plaque sample)			*p*-Value	Allele change (Frequency) Mean ± Standard deviation (Blood sample)			
	AG	GC	CT		AG	GC	CT	*p*-Value
Age (years)	56.2 ± 13.3	50.6 ± 11.2	38 ± 12.4	0.01†	52.2 ± 9.9	46.6 ± 7.8	34 ± 9	0.01†
Height (cm)	174.2 ± 16.1	168.7 ± 11	172.2 ± 9.1	0.963[NS]	163.2 ± 6.7	164.7 ± 7.8	167 ± 5.7	0.806[NS]
Weight (Kg)	74.8 ± 20.4	66.6 ± 6.1	67.8 ± 8.9	0.321[NS]	68.8 ± 9	60.6 ± 2.5	63.8 ± 3.5	0.24[NS]
Body Mass Index (BMI)	32.2 ± 10.6	26.5 ± 7.3	30.4 ± 4.8	0.114[NS]	25 ± 3.2	22.5 ± 1.9	23 ± 1.4	0.101[NS]
Waist Hip ratio	4.9 ± 3.4	2.8 ± 3.4	4.8 ± 3.4	0.437[NS]	0.9 ± 0.04	0.8 ± 0.03	0.8 ± 0.03	0.435[NS]
Monthly Income (Rs)	285.71 ± 7204.4	22600 ± 7916.7	28500 ± 8888.2	0.447[NS]	20769.23 ± 6002.13	31666.67 ± 5773.503	20076.92 ± 7750.930	0.1[NS]
Plaque Index	6.4 ± 3.7	5.7 ± 7.5	4.7 ± 3.5	0.05*	0.8 ± 0.3	0.7 ± 0.1	0.7 ± 0.1	0.05*
Bleeding on Probing (%)	75.7 ± 29.8	77 ± 8.6	87 ± 8.2	0.05*	71.7 ± 20.4	73 ± 5.2	78.2 ± 4.8	0.627[NS]
Probing Pocket Depth (mm)	7.9 ± 4.3	10.3 ± 2.8	8.2 ± 3.7	0.67[NS]	3.9 ± 0.9	3.9 ± 0.2	4.2 ± 0.3	0.05*
Clinical Attachment Level (mm)	8 ± 4.4	8.7 ± 2.9	8.6 ± 3.6	0.01†	5 ± 1	4.7 ± 0.3	4.6 ± 0.2	0.01†
Total Cholesterol (mg/dl)	192.4 ± 41.6	186.4 ± 28.6	140.4 ± 30.2	0.321[NS]	180.4 ± 38.2	182.4 ± 24	136 ± 26.8	0.13[NS]
High-Density Lipoprotein (mg/dl)	39.3 ± 12.6	45.3 ± 11.9	64.8 ± 7.5	0.197[NS]	35.3 ± 7.2	41.3 ± 5.5	40.8 ± 4.1	0.2[NS]
Low-Density Lipoprotein (mg/dl)	130.5 ± 41.8	115.4 ± 29.6	89.8 ± 12.2	0.05*	126.5 ± 38.4	111.4 ± 21.2	85.8 ± 8.8	0.146[NS]
Triglycerides (mg/dl)	129.3 ± 37.4	170.4 ± 38.1	143.6 ± 38.2	0.1[NS]	125.3 ± 34	162.4 ± 33.3	133 ± 34.8	0.268[NS]
Systolic Blood Pressure (mm Hg)	130 ± 15.5	128.3 ± 10.7	121.1 ± 8.4	0.1[NS]	129 ± 12.1	124.3 ± 5.3	117.5 ± 5	0.1[NS]
Diastolic Blood Pressure (mm Hg)	84.5 ± 11.9	78.3 ± 8.7	79.4 ± 9.2	0.1[NS]	80.5 ± 5.1	74.3 ± 5.3	75 ± 5.8	0.1[NS]

* $p < 0.05$ is considered to be statistically significant; † $p < 0.01$ is considered to be highly statistically significant; ‡ $p<0.001$ is considered to be very highly statistically significant; § NS—Non significant.

Table S2. Overall comparison of the demographic variables, periodontal parameters and cardiac parameters with the allele change (frequency) of the CARD8 (rs2043211) in subgingival plaque and blood samples for both the groups.

Variable	Allele change (Frequency) Mean ± Standard deviation (Subgingival plaque sample)			*p*-Value	Allele change (Frequency) Mean ± Standard deviation (Blood sample)			
	GA	AC	TC		GA	AC	TC	*p*-Value
**Age (years)**	57 ± 7.6	42.5 ± 8.4	52.9 ± 7	0.001‡	51.4 ± 9.6	48.6 ± 9.7	51.6 ± 14.5	0.648^{NS}
**Height (cm)**	164.4 ± 5.2	163.8 ± 6.2	166.4 ± 11	0.867^{NS}	161.7 ± 6.4	168.4 ± 9.8	163.3 ± 6.1	0.05*
**Weight (Kg)**	68.2 ± 6.3	67 ± 8.1	69.8 ± 10.6	0.513^{NS}	68.4 ± 6.6	67.1 ± 12.6	63.6 ± 3.9	0.379^{NS}
**Body Mass Index (BMI)**	25.3 ± 3.3	25.1 ± 3.7	23.8 ± 2.4	0.262^{NS}	25.7 ± 3.2	23.7 ± 2.8	24 ± 2.3	0.01†
**Waist Hip ratio**	0.8 ± 0.1	0.8 ± 0.03	0.8 ± 0.02	0.793^{NS}	0.9 ± 0.1	0.8 ± 0.03	0.8 ± 0.02	0.368^{NS}
**Monthly Income (Rs)**	25500 ± 8812.9	24071.43 ± 8416.7	16700 ± 3772.7	0.11^{NS}	22900 ± 6719.2	23000 ± 8932.5	20727.3 ± 8088.2	0.714^{NS}
**Plaque Index**	0.9 ± 0.1	0.7 ± 0.1	1 ± 0.2	0.001‡	0.7 ± 0.2	0.8 ± 0.2	0.9 ± 0.2	0.05*
**Bleeding on Probing (%)**	71.4 ± 9.6	74.4 ± 2.9	75.1 ± 27	0.715^{NS}	68.9 ± 19.3	74.5 ± 18.6	77.2 ± 25.5	0.516^{NS}
**Probing Pocket Depth (mm)**	4.6 ± 0.2	3.1 ± 0.9	4.7 ± 0.3	0.001‡	3.7 ± 0.9	4.3 ± 0.4	4.6 ± 0.3	0.01†
**Clinical Attachment Level (mm)**	5.6 ± 0.4	4.3 ± 0.4	5.9 ± 0.6	0.001‡	4.6 ± 0.9	5.3 ± 0.6	5.7 ± 0.8	0.01†
**Total Cholesterol (mg/dl)**	181.4 ± 27	159.3 ± 6.3	194.3 ± 33.4	0.522^{NS}	180.6 ± 30.4	176.4 ± 32.8	178.4 ± 71.1	0.952^{NS}
**High-Density Lipoprotein (mg/dl)**	35.1 ± 3.2	39.8 ± 6.6	36 ± 9.6	0.344^{NS}	38.5 ± 7.2	37.6 ± 8.7	39.3 ± 9.3	0.860^{NS}
**Low-Density Lipoprotein (mg/dl)**	122.8 ± 20.7	97.5 ± 9.3	134.3 ± 33.7	0.146^{NS}	119.9 ± 33.3	115.7 ± 33.6	118.5 ± 45.6	0.934^{NS}
**Triglycerides (mg/dl)**	120.8 ± 32.4	141.5 ± 18.9	171.2 ± 66.8	0.303^{NS}	124.4 ± 40.5	144.7 ± 60	130.1 ± 29.9	0.392^{NS}
**Systolic Blood Pressure (mm Hg)**	124.3 ± 11.3	125 ± 5.8	128 ± 10.3	0.1^{NS}	130 ± 10.8	123.5 ± 5.9	120.9 ± 12.2	0.05*
**Diastolic Blood Pressure (mm Hg)**	79.3 ± 4.9	72.5 ± 5	82 ± 6.3	0.05*	79.5 ± 6	79.5 ± 6	79.1 ± 5.4	0.980^{NS}

* $p < 0.05$ is considered to be statistically significant; † $p < 0.01$ is considered to be highly statistically significant; ‡ $p<0.001$ is considered to be very highly statistically significant; § NS—Non significant.
